# STSE: Spatio-Temporal Simulation Environment Dedicated to Biology

**DOI:** 10.1186/1471-2105-12-126

**Published:** 2011-04-28

**Authors:** Szymon Stoma, Martina Fröhlich, Susanne Gerber, Edda Klipp

**Affiliations:** 1 Humboldt-Universität zu Berlin, Department of Theoretical Biophysics, Invalidenstr. 42, Berlin, Germany; 2 Università della Svizzera italiana (USI), Institute of Computational Science, Computational Time Series Analysis, Via Giuseppe Buffi 13, 6900 Lugano, Switzerland

## Abstract

**Background:**

Recently, the availability of high-resolution microscopy together with the advancements in the development of biomarkers as reporters of biomolecular interactions increased the importance of imaging methods in molecular cell biology. These techniques enable the investigation of cellular characteristics like volume, size and geometry as well as volume and geometry of intracellular compartments, and the amount of existing proteins in a spatially resolved manner. Such detailed investigations opened up many new areas of research in the study of spatial, complex and dynamic cellular systems. One of the crucial challenges for the study of such systems is the design of a well stuctured and optimized workflow to provide a systematic and efficient hypothesis verification. Computer Science can efficiently address this task by providing software that facilitates handling, analysis, and evaluation of biological data to the benefit of experimenters and modelers.

**Results:**

The Spatio-Temporal Simulation Environment (STSE) is a set of *open-source *tools provided to conduct spatio-temporal simulations in discrete structures based on microscopy images. The framework contains modules to *digitize, represent, analyze*, and *mathematically model *spatial distributions of biochemical species. Graphical user interface (GUI) tools provided with the software enable meshing of the simulation space based on the Voronoi concept. In addition, it supports to automatically acquire spatial information to the mesh from the images based on pixel luminosity (e.g. corresponding to molecular levels from microscopy images). STSE is freely available either as a stand-alone version or included in the linux live distribution Systems Biology Operational Software (SB.OS) and can be downloaded from http://www.stse-software.org/. The Python source code as well as a comprehensive user manual and video tutorials are also offered to the research community. We discuss main concepts of the STSE design and workflow. We demonstrate it's usefulness using the example of a signaling cascade leading to formation of a morphological gradient of Fus3 within the cytoplasm of the mating yeast cell *Saccharomyces cerevisiae*.

**Conclusions:**

STSE is an efficient and powerful novel platform, designed for computational handling and evaluation of microscopic images. It allows for an uninterrupted workflow including digitization, representation, analysis, and mathematical modeling. By providing the means to relate the simulation to the image data it allows for systematic, image driven model validation or rejection. STSE can be scripted and extended using the Python language. STSE should be considered rather as an API together with workflow guidelines and a collection of GUI tools than a stand alone application. The priority of the project is to provide an easy and intuitive way of extending and customizing software using the Python language.

## Background

With the availability of high-resolution microscopy and high-throughput technologies in molecular biology the amount of cellular images in very good resolution quality increases significantly. Such amount of available data consecutively demands for image analysis software adapted to utilize the full capacity of these imaging advancements. The state-of-the-art way of presenting, assessing and evaluating experimental images qualitatively is being increasingly replaced by computational data evaluation. Quantification of e.g. light intensities arising from fluorescent protein (FP) expression in different cellular compartments can be ascertained in a spatially resolved manner and enables us to mathematically verify the current understanding of biological systems.

Unambiguous and reproducible computational extraction increases the quality and exchangeability of information for subsequent automatic processing steps such as digitization, representation, analysis, and modeling. A variety of image processing-, analysis- or modeling-packages addressing these tasks exist already, either on a commercial basis or as open source software.

Recently, several eminent reviews have been published which outline the most common methods and tools addressing biological image processing, analysis and modeling (see [1-3]). One of the key conclusions is that these tasks are usually separately addressed. Cell segmentation and property extraction, for example, are well established and can be realized by dedicated software such as CellProfiler [4], Cell-ID [5] or generic image processing platforms like Labview (National Instruments, Austin, USA) or Imaris (Bitplane, Zurich, Switzerland). A widely used and freely available tool is ImageJ [6], which comprises standard segmentation algorithms as well as surface or profile plots. Also freely available are additional packages for R like EBImage [7], which can be used for the segmentation and analysis steps. When it comes to spatial modeling and simulation in microbiology one can distinguish the following classes of dedicated simulators i) spatially partitioned ODE systems (e.g. Virtual Cell) ii) spatially partitioned Gillespie systems (e.g. MesoRD [8], SmartCell [9]) iii) particle-based simulators (e.g. Smoldyn [10], MCell [11], Meredys [12]). These techniques differ mostly with respect to the mathematical framework which changes the level of detail represented in the system (e.g. spatially partitioned ODEs are giving the overview of the system and can be used at tissue scale and large time scale, whereas particle based simulators are able to represent the molecule-scale details, however time scale needs to be importantly shortened).

**Figure 1:**
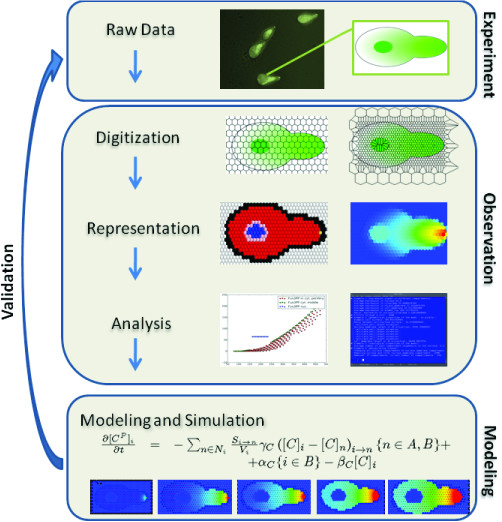
**Flow Chart**. Sketch of the structure and the modules combined in STSE that allow for an uninterrupted workflow. Starting with microscopy images (raw data) the framework allows for digitize, represent, analyze and mathematically model spatial distributions of species.

All of these tools offer excellent solutions for the specific problems they were designed to solve. However, it is still difficult to perform a contiguous and intuitive workflow, starting with almost raw data images and resulting in a running mathematical model, that enables to directly compare the simulation results with biological data (as presented in Figure 1).

The STSE platform intends to close the gaps between *various *tools or software-packages that are in majority specifically designed for these separate steps (i.e image processing/analysis or modeling/simulation). By providing the workflow guidelines and the access to Python language, it offers the advantage of stratifying the interaction with different data-structures and thus minimizes the loss of time and information during the manual export and conversion processes. Therefore, it should be seen as a set of tools facilitating the intuitive workflow between the image analysis tools and simulators.

Additionally, in its current implementation, it provides examples of how to perform such a transition from segmenting tools to simulation engines implemented internally in Python (spatially partitioned ODEs). For these purposes STSE comprises modules for digitizing and representing microscopy data, enables data analysis as well as manipulation, and can be used for mathematical modeling and simulation of spatial distributions of chemical species. It is a powerful, multifaceted tool for interdisciplinary work.

## Implementation

The tools are written in Python and have a modular design which allows the modeler to extend their functionality according to custom needs. The default STSE workflow can be summarized as follows (see: Figure 1):

1. Preprocessing of microscopic images for the studied object.

2. Definition of a discrete representation of the images.

3. Automatic integration of the information from images into the discrete representation.

4. Analyzing the digitized data.

5. Formulating a model: defining interactions between regions of interest and molecules of interest.

6. Running a model: previously digitized images are used as initial conditions for the evaluation of simulation results.

A detailed use case as well as comparative studies with some of the above mentioned state-of-the-art tools is provided in the additional file 1. Additionally, the webpage of the project contains examples, video tutorials, access to a discussion group and other helpful information sources. In the following, we give a concise overview of the fundamental methods used in STSE:

### Spatial Segmentation and Digitization

The process of digitization generates a data structure, allowing for efficient analysis, representation and modeling. The classical approach is to decompose the microscopy image into physiologically distinguishable compartments (e.g. nucleus, cytoplasm, etc.) which is called image segmentation [13,14]. Usually, image segmentation results in a data structure linking the compartments with pixels. STSE differs from this approach by introducing an abstract, intermediate layer composed of so-called subcompartments. To generate this layer, each compartment is divided into subcompartments which have the geometry of polygons and are organized in such a way that they fill the entire compartment and do not overlap with each other. The default geometry is automatically composed of equilateral hexagons. The purpose of introducing this abstract layer is to allow for adjusting the digitization precision separately for different compartments, which is useful when it comes to analysis and modeling. To edit the geometry of subcompartments a Voronoi 2D tessellation is used [15]. With the help of the graphical user interface (GUI) editor, the user may move subcompartment centers (corresponding to the vertices in the Voronoi graph) for fine-tuning. This information implicitly specifies the geometry of each subcompartment. Since these subcompartments share edges, the representation resembles a polygonal mesh (PM).

Each subcompartment has an individual geometry as well as other user-customizable properties such as cellular compartment affiliation, concentrations of specific substances, etc. The GUI allows for user-friendly inspecting and editing of these properties. Additionally, due to the software implementation design, it is possible to extend the GUI editor by adding custom actions as well as to script the GUI with Python. One of the main goals of STSE is to provide the possibility of framework extension and customization to the users.

With STSE it is possible to acquire spatial luminosity information from microscopic images, which can correspond e.g. to the inhomogeneous distribution of tagged molecules within the cell. This process is performed on indexed color images (e.g. FP microscopic images). This is an important feature, since it allows for the comparison of simulation results with experimental data.

### Representation and Analysis

Image representation is performed implicitly by the conversion of the Voronoi-based PM to an internal STSE data structure. This design involves less constraints and thus allows for more latitude in defining polygonal geometries (e.g. including non-convex ones) as well as physiological information. It is realized by storing the polygon corner coordinates explicitly in the data structure instead of computing them using the Voronoi algorithm. The datastructure may be easily modified or inspected via Python. This allows for simulating structures changing in time, which has been, for instance, successfully used in the dynamic modeling of meristem growth [16]. The analysis is effected via the STSE-GUI as well as with Python scripts and enables a comprehensive and differentiated overview of topological, geometrical and physiological information. The routines provided by STSE allow for visualizing and inspecting compartment properties and can be used for computing different properties and for further, computational analysis of data from images. All structural information can be exported and saved for persistence and dissemination.

### Modeling

The digitized data can be used *directly *to perform spatial modeling (e.g. as initial conditions or evaluation). STSE does not restrict the user with the simulation framework. Instead, we suggest a workflow based on the so-called "cell-centered" finite volume method [17]. According to this scheme, a mechanistic model of a studied process needs to be formalized using a set of ordinary differential equations (ODEs) describing the interplay of different actors (e.g. chemical molecules) and different cellular compartments with specified kinetic rules on diffusion, chemical reactions, transport, etc. In this case a SciPy library [18] may be used to solve the system within the STSE framework.

Due to its design STSE is fully extendable via Python. The simulation engine can be freely connected with multitude of solutions limited only by the accessibility of these engines via Python.

## Results

In the following, we demonstrate how to use STSE to analyze and simulate biological systems. A typical STSE workflow includes the modules for digitization, representation, analysis and modeling is presented using the running example of a mitogen-activated protein kinase gradient formation (the double-phosphorylated Fus3 (*Fus3^PP^*) in a mating yeast cell [19].

Fus3 signaling is part of the yeast mating pheromone pathways: upon stimulation with the pheromone *α*-factor, an intracellular signaling cascade becomes activated, which leads to the double phosphorylation of Fus3. The *Fus3^PP ^*gets released at the shmoo tip and can diffuse within the cell, which results in an observable *Fus3^PP ^*gradient. When reaching the nucleus, *Fus3^PP ^*is actively transported across the nuclear membrane and regulates transcription factors that modulate mating-specific gene expression. We would like to stress that the focus is set rather on the software specifications and the application scenario than on the biological results. To simplify the analysis and to facilitate the usage of examples in a confirmatory way, we work on test data, inspired by the experiments and explanations presented by Maeder et al. [19].

An extended workflow comprising amongst others the following examples is given and discussed in the additional file 1. Additionally, video tutorials covering this subject and all Python scripts we use to produce the here presented images and results are provided on the project homepage.

In a first step we demonstrate the analysis and characterization of the *Fus3^PP ^*gradient. For this purpose we:

• Quantify the ratio of the average cytoplasm/nucleus expression of *Fus3^PP ^*based on fluorescence signal intensity acquired from microscopy images,

• Show gradient curves for *Fus3^PP ^*along the x-axis of the cell data image and around the nucleus,

• Simulate the process of *Fus3^PP ^*diffusion in the cytoplasm to determine the underlying conditions that lead to the qualitative values captured in the image.

We evaluate the results of the simulations and discuss i) whether the appearance of a *Fus3^PP ^*gradient throughout the cell can be explained by simple diffusion and ii) how to define plausible conditions and model parameters allowing to reproduce the experimental observations.

### Digitization

**Figure 2:**
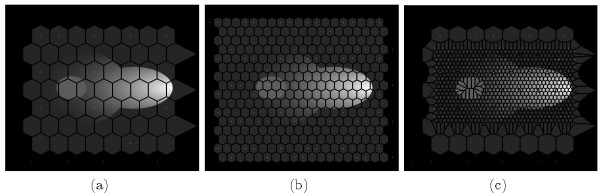
**Subcompartment Densities**. Different "subcompartment density" variants (a) rough regular digitization (b) more refined, regular digitization (c) refined, irregular representation edited with the STSE GUI.

**Figure 3:**
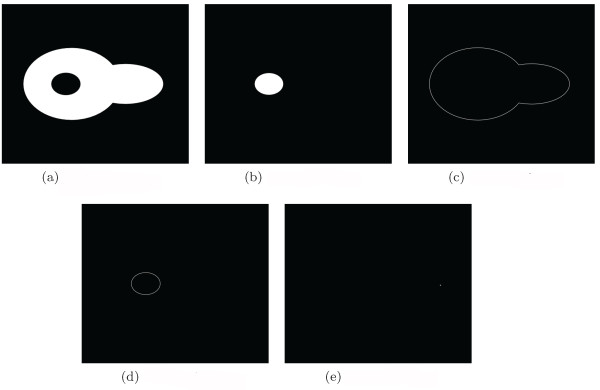
**Binary Masks**. Binary masks created from raw data images. The images present the binary masks for (a) the cytoplasm, (b) the nucleus, (c) the cell membrane, (d) the nuclear membrane and (e) the shmoo tip (a single pixel is sufficient to mark the shmoo tip).

**Figure 4:**
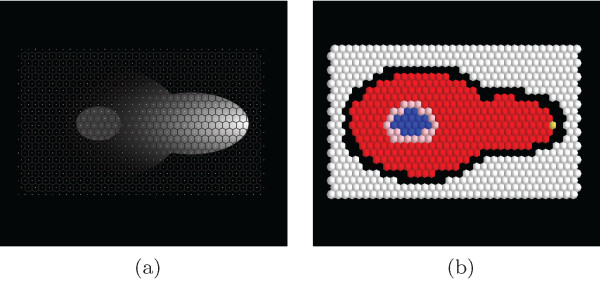
**Subcompartment Type Assignment**. Subcompartment types assignment (a) mesh showing the geometry of subcompartments (b) types of subcompartments acquired from the binary masks. Different sphere colors depict different compartment identities: white - outside, black - the cell membrane, blue - the nucleus, red - the cytoplasm, pink - the nuclear membrane, yellow - the shmoo tip.

A major issue in this context is the task to adapted the polygonal mesh. If, for instance the focus is on a particular protein like the *Fus3^PP ^*in this case, the interesting point is the protein gradient within the cytoplasm but not outside the cell. Thus it is necessary and sufficient to adapt the mesh size according to the area of interest. Here, it is requested to keep a high precision within the cytoplasmic compartment (but not within other compartments) in order to capture and depict the gradient correctly. The analysis accounts the hypothesis that the *Fus3^PP ^*distribution is neither outside the cell nor in the nucleus (motivation for this is discussed in the additional file 1). Therefore, we use varying "subcompartment densities" in these compartments as presented in Figure 2. The default geometry is automatically composed of equilateral hexagons (see Figure 2a and 2b). The geometry of the subcompartments can afterward be fine-tuned using the GUI editor to match different analysis and modeling requirements (see Figure 2c). Another task related to the digitization of image data is the acquisition of subcompartment types (i.e. determining for each abstract subcompartment its affiliation to a cellular compartment). This task can be performed via the GUI or a Python script. Although a subcompartment type can be set manually, in both cases the recommended way is to use an automatic protocol based on binary masks. These binary masks are based on original microscopy images and can be prepared with 3rd party segmentation algorithms (e.g. implemented in ImageJ). Each subcompartment is associated with only one compartment type. When a conflict occures (e.g. in the case of overlapping binary masks) the user can influence subcompartment types by changing the order of application of the binary masks or by defining subcompartment types manually. Here, we use binary masks for localization of the following cell types (see Figure 3): the cytoplasm (3a), the nucleus (3b), the cell membrane (3c), the nuclear membrane (3d) and the shmoo tip (3e). These mask files are used to acquire the subcompartment types either by GUI (Figure 4) or a Python script. Both methods are covered in detail in the additional file 1.

**Figure 5:**
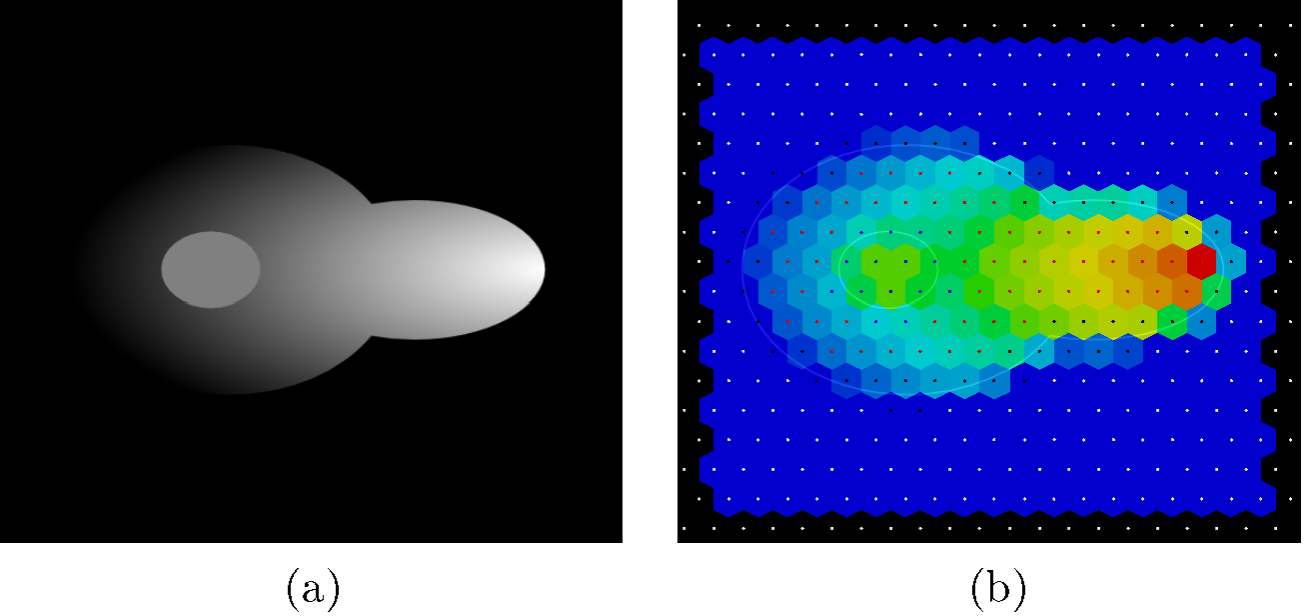
**Signal Quantification**. Signal quantification (a) indexed color image of Fus3^PP ^localization (b) result of Fus3^PP ^signal quantification based on the indexed color image. Small spheres depict the compartment types.

The automatic acquisition of the signal from the microscopy image is another demanding task and provides the basis for the subsequent analysis and modeling steps. For this purpose we use indexed color images (e.g. standard light/confocal microscopy images) corresponding to molecular concentrations of the molecules of interest. In the running example we use test data images inspired by the experiments described in Maeder et al. [19], in which the intracellular localization of *Fus3^PP ^*has been reported by fluorescence lifetime imaging microscopy (FLIM) (see Figure 5). In this particular case, since we are interested only in one chemical species (*Fus3^PP^*), for each time step we provide only one image (corresponding to a specific channel in fluorescent microscopy). In the more general case, a number of images required for each acquisition time step ideally should be equal to the number of species of interests. To summarize the previous steps: The necessary inputs for the digitization procedure are i) the binary masks, and ii), the indexed color images. The output of the digitization step is a feasible amount of abstract sub-compartments that cover the microscopic image. Each subcompartment is allocated with a specific compartment type and the average intensity of the protein(s) of interest acquired from input data.

### Representation and analysis

**Figure 6:**
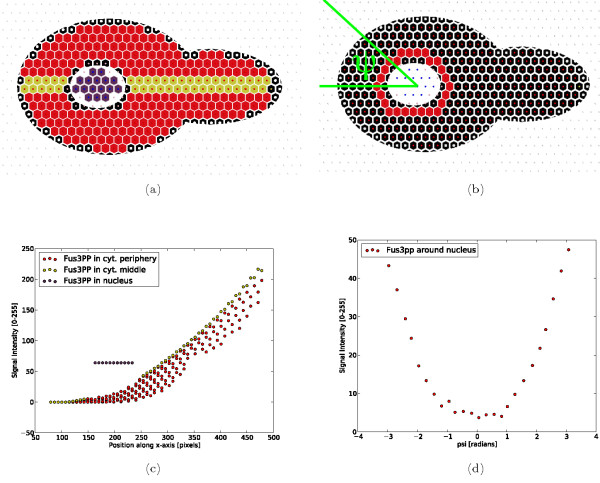
**Fus3 Profile**. Fus3^PP ^profiles along the *x*-axis and around the nucleus. (a) subcompartment locations used to distinguish the curves in (c); (b) subcompartment locations and *ψphi *definition used in (d); (c) Fus3^PP ^profiles along the *x*-axis; (d) Fus3^PP ^profiles around the nucleus.

The analysis in STSE is realized via Python scripts. Our running example demonstrates common tasks performed with STSE such as inspecting geometrical, physiological or topological properties of the subcompartments/compartments and removing or resizing the subcompartments. The following information on the *Fus3^PP ^*gradient can be extracted (see Figure 6):

• The distribution of *Fus3^PP ^*in the cytoplasm along the x-axis in a central part of the analyzed cell is exponential (Figure 6c),

• The distribution of *Fus3^PP ^*around the nucleus reaches its maximum in the point closest to the shmoo tip (Figure 6d),

• The average *Fus3^PP ^*signal in the nucleus is 64.0 (a.u.), which is ≈ 25% of the maximal signal measured in the image,

• The average *Fus3^PP ^*signal in the cytoplasm is 52.07 (a.u.) (which is ≈ 20% of the maximal signal measured in the image),

• The ratio of averaged *Fus3^PP ^*signal in the cytoplasm to nucleus is equal to 0.81.

Comparative values computed with ImageJ showed an average *Fus3^PP ^*signal in the nucleus of 64.0 (a.u.) and an average *Fus3^PP ^*signal in the cytoplasm of 51.27 (a.u.), resulting in a ratio of averaged *Fus3^PP ^*signal in the cytoplasm to nucleus of 0.801. Which means the relative error due to approximation (i.e. downsampling because of using averaged values from PM instead of actual pixel values) in our example is (0.81 - 0.801)/0.801 ≈ 1%.

### Modeling

The previously acquired, quantified and structured data can be used to create a dynamic model of the *Fus3^PP ^*diffusion. According to the STSE dataflow paradigm (see Figure 1), the mechanistic model of the studied process needs to be formalized. By focusing on the properties of the *Fus3^PP ^*gradient we can exclude processes such as i) mechanisms of the stimulation of Fus3 and ii) different mechanisms allowing *Fus3^PP ^*to enter the nucleus. The kinetic model of *Fus3^PP ^*is now defined as follows:

• *Fus3^PP ^*appears in the shmoo tip compartment,

• *Fus3^PP ^*diffuses freely in the cytoplasm compartment,

• *Fus3^PP ^*gets dephosphorylated during the diffusion in the cytoplasm,

• *Fus3^PP ^*is unable to cross the cellular/nuclear membrane compartments.

By applying this kinetic model it is next possible to verify whether or not the qualitative properties of the *Fus3^PP ^*gradient observed in the digitized images can be reproduced. As explained in the Background section, STSE functionality can be modified and extended by connecting various simulation engines via Python. Here, for the purpose of simplification, we use a dedicated, explicit simulation, written directly in Python. For this purpose the model is translated into a system of differential equations (for details please see the additional file 1). An equation describing the changes of *Fus3^PP ^*concentration is attributed to each subcompartment.

where:

• *FUS3^PP^_i _*is the concentration of Fus3^PP ^in the subcompartment *i*,

• γ *_FUS3_^PP ^*is the diffusion constant for Fus3^PP^,

• α*_FUS3_^PP ^*is the rate of Fus3^PP ^release in the shmoo tip,

• β*_FUS3_^PP ^*is the rate constant of Fus3^PP ^dephosphorylation,

• *S*_*i*→*n *_is the area of contact surface between subcompartments *i *and *n*,

• *V_i _*is the volume of subcompartment *i*,

• *i *∈ *A */*i *∈ *B *if *i *belongs to cytoplasm/shmoo tip compartment,

• *N_i _*is a set of neighbour subcompartments for subcompartment *i*,

• , (e.g. [*n *∈ *A *∪ *B*] evaluates to 1 when *n *is element of *A *or *B*) [20,21].

To complete the model it is required to define the rate of Fus3^PP ^release in the shmoo tip (*α*_*FUS3*_^*PP*^), the rate constant of Fus3^PP ^dephosphorylation *β*_*FUS*3_^*PP*^, the diffusion constant *γ*_*FUS3*_^*PP *^and the initial conditions. All values can be estimated from the literature or chosen arbitrarily. Additionally, initial conditions can be acquired from the digitization step of the image data. An exemplary implementation of the *Fus*3*^PP ^*model can be downloaded from the project homepage. An animation showing the kinetics of *Fus*3*^PP ^*distribution obtained with the implemented model is available as additional file 2.

**Figure 7:**
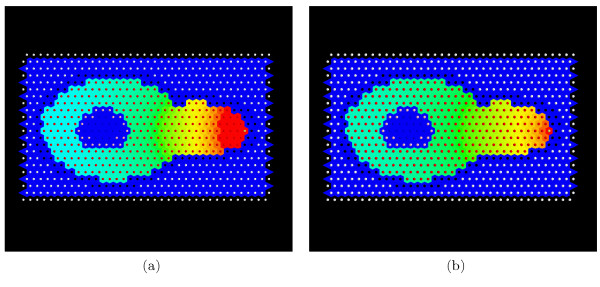
**Steady State Distribution**. Steady state distributions of Fus3^PP ^for two different parameter sets (*α*_*Fus*3_, *β*_*Fus*3_, *γ*_*Fus*3_): (a) (0.1, 0.1, 100), (b) (0.1, 0.1, 50). We observe that the gradients have different slopes, which is due to the difference in the diffusion constant *γ*_*Fus*3_. To visualize Fus3^PP ^concentrations, a colormap is used where blue depicts low values and red depicts high values.

Simulations in STSE can also be utilized to estimate the values of *Fus*3*^PP ^*model parameters based on the image data. As an example, the steady state concentrations for two different simulations are presented in Figure 7 (for details see additional file 1).

**Figure 8:**
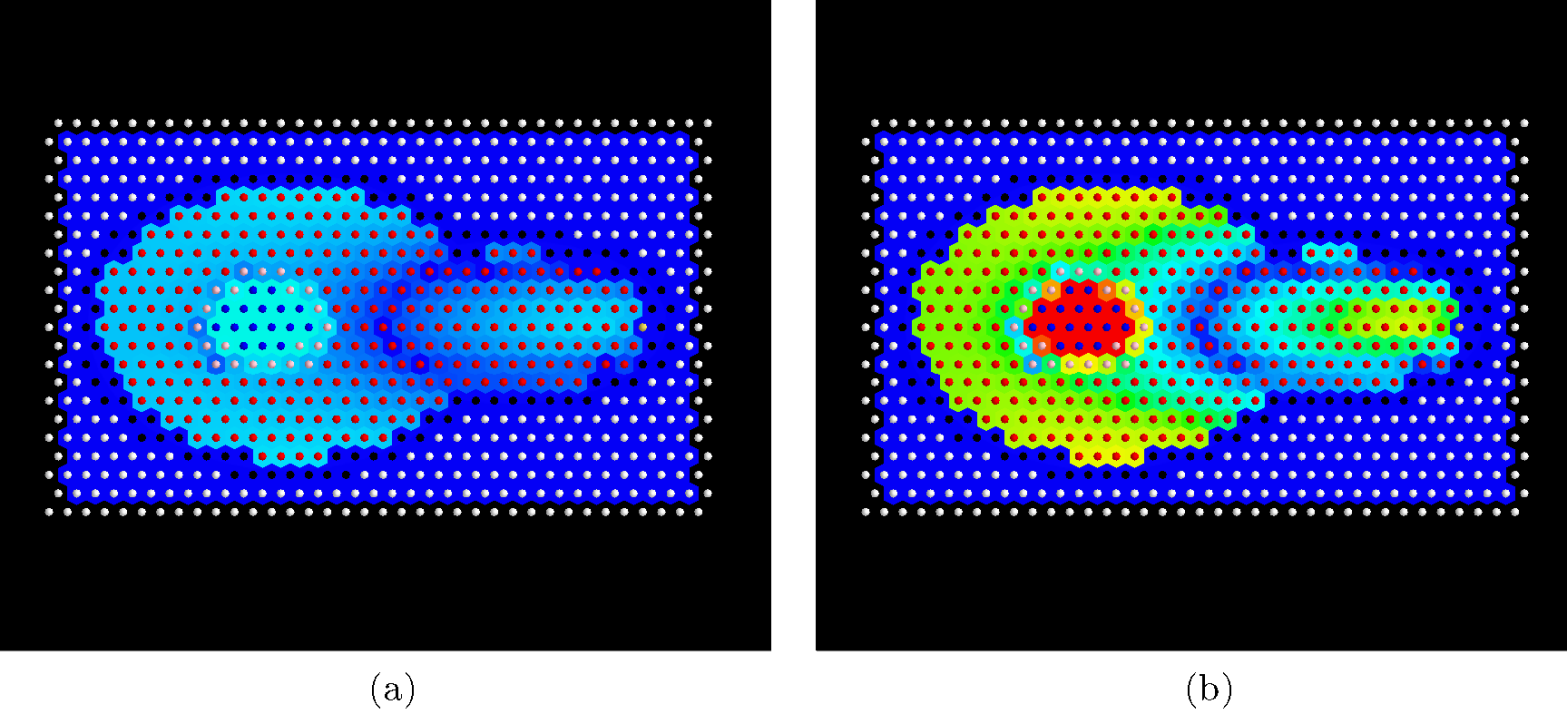
**Difference Concentration**. Difference of Fus3^PP ^concentration between  and (which approximates the error). (a) shows an  (the overall error did not exceed 20% percent) (b) shows the *E*/*max*(*E*) (when 100% of error was observed in the center; it is important to note, that the model did not allow the Fus3^PP ^to enter the nucleus compartment). To visualize *E*, a colormap is used where blue depicts low values and red high ones.

A second animation (additional file 3) shows the kinetics of *Fus*3*^PP ^*evolution. This simulation differs by changing the diffusion constant for Fus3^PP ^from 50 to 100. For further illustration, Figure 8 presents the contrast between different initial parameter sets. This difference can be used to discriminate between different parameter sets.

## Discussion

We present STSE, a platform that facilitates execution of spatial simulations based on microscopy images. The application of the STSE is demonstrated on the example of the yeast pheromone MAP kinase cascade, focusing in particular on the distribution of the double-phosphorylated Fus3.

We demonstrate how to quantify the ratio of the average cytoplasm/nucleus expression of *Fus*3*^PP ^*based on fluorescence signal intensity acquired from microscopy images, create gradient curves for *Fus*3*^PP ^*along the x-axis of the cell and around the nucleus, and simulate the process of *Fus*3*^PP ^*diffusion in the cytoplasm to determine the underlying mechanism.

The result of the simulations allow us to confirm that a set of hypothesis used in the model allows us to reproduce the experimental observations. We demonstrate also how to use the STSE to discriminate between model parameter sets.

The running example model yields the highest error in the nuclear compartment. This is consistent with our expectations: the test data set images suggest that *Fus*3*^PP ^*is present in the nuclear compartment (Figure 4), but in the specification of the model we skipped the mechanism of *Fus*3*^PP ^*transport via membranes, which results in the *Fus*3*^PP ^*concentration in the nuclear compartment being equal to 0. To correct this property, the model should be extended by an assumption of *Fus*3*^PP ^*transport via the nuclear membrane.

In the additional file 1 STSE modules are also compared with a selection of other available software tools which allow to perform each of the workflow substeps separately i.e. digitization, representation, analysis or modeling.

Results from STSE are confronted with results achieved by the use of the software ImageJ. Both tools allow for computing the ratio between the *Fus*3*^PP ^*signal in the cytoplasm and the nucleus. Although STSE uses the approximation with subcompartments (which are very usefull when it comes to the simulation task) the approximation error in this example is below 1%. Likewise, the exponential decrease of Fus3^PP ^along the cell center as well as the increase in the nucleus can be captured reasonably well with both tools. Nevertheless, if one is interested in the distribution of Fus3^PP ^over the whole cell, there is no constitutive way to do so in ImageJ (however plugins allow to perform similar actions). Furthermore, in STSE it is possible to plot various profiles for any selected subcompartments.

In ImageJ basic analysis is performed via the GUI, but an extensive analysis again requires the usage of Macros, Plugins or Scripts (via Java-like, Java or JavaScript). STSE requires using Python in both basic and extensive analysis. In the latter case the automatization of tasks via Python allows for faster implementation.

Binary masks can be used for automatization in STSE. The choice of the segmentation algorithm to generate those binary masks depends highly on the particular problem and there already exist a multitude of advanced software packages dedicated to this task. Therefore in the current version of the STSE we decided to use already segmented images as the starting point of the proposed workflow and leave the choice of the optimal segmentation method to the user.

STSE conceptually differs from selection-based tools by operating on abstract subcompartments rather than on pixels directly. This approach results in the simplification of further processing and it allows for inhomogenous precision in different cellular compartments.

The precision is regulated in STSE by decreasing the subcompartment size. However, the increase in precision induces slower execution of the modeling routines. It is the modeler's choice to prepare the grid in such a way that both, the precision and speed of modeling routines is optimal. Also it should be kept in mind that, by using larger subcompartments, the assumption of homogeneously distributed molecules within one subcompartment might be disregarded.

In the additional file 1 we also compare STSE with a selected simulation engine, MesoRD [8], and discuss the main differences, pros and cons. It is important to understand that the scopes of these softwares are different: MesoRD is a mesoscopic, stochastic simulation engine, whereas STSE covers much broader area, but it does not provide a sophisticated simulation engine as such. Our future goal is to provide integration of STSE with a selection of existing simulation software. This would allow users to use state-of-the-art solutions for simulation, keeping the ease of validating the models with microscopy data, as demonstrated in our example.

## Conclusion

STSE is a software platform, designed for constructing microscopy image-based simulations. It allows for an uninterrupted workflow including digitization, representation, analysis, and mathematical modeling. The main benefit of STSE is that it acts as a "glue" between different steps of the workflow, allowing the user to tailor the platform to their specific needs. Due to its open, modular architecture and integration of Python language, the software allows for full automatization (it applies also to GUI) via scripts, which is usually not possible or very limited with other stand alone applications.

Further versions of the STSE should provide integration of selected 3rd party simulators and simulation paradigms (e.g. stochastic, agent-based). It would be also crucial to support import and export of SBML (Systems Biology Markup Language) files. For the latter, a prior establishment of a standard for spatial modeling would be required. We strongly encourage the community to provide examples of various microscopy based simulation workflows, which we would be glad to integrate into STSE framework.

## Availability and requirements

**Project name: **STSE

**Project home page**: http://stse-software.org

**Operating system(s**): Linux (availability on other systems depends on 3rd party libraries)

**Programming language**: Python

**Other requirements: **Openalea http://openalea.gforge.inria.fr/, Mayavi2 http://code.enthought.com/projects/mayavi/, Qhull http://www.qhull.org/, NetworkX http://networkx.lanl.gov/. It is also possible to use the software directly from a live DVD Linux distribution, SB.OS http://www.sbos.eu/, which comes with a comprehensive list of other systems biology software.

**License: **GNU GPL

## Authors' contributions

SS is the main developer of STSE. MF, SG, SS, EK wrote the article. MF, SG, SS applied the STSE workflow to the example described in the Results and performed comparative studies of STSE and other state-of-the-art tools. All authors read and approved the final manuscript.

## Supplementary Material

Additional file 1 **Detailed description of a use case, including all individual steps of the STSE workflow with examples as well as comparative studies with state-of-the-art tools**. Click here for file

Additional file 2 **Animation showing the dynamics of the exemplary system described in the additional file **1. Click here for file

Additional file 3 **Animation showing the dynamics of the exemplary system described in the additional file **1. Click here for file

## References

[B1] Ljosa V Carpenter AE Introduction to the Quantitative Analysis of Two-Dimensional Fluorescence Microscopy Images for Cell-Based Screening PLoS Comput Biol 2009 5 12 e1000603 10.1371/journal.pcbi.1000603 20041172 PMC2791844

[B2] Peng H Bioimage informatics: a new area of engineering biology Bioinformatics 2008 24 17 1827 1836 10.1093/bioinformatics/btn346 18603566 PMC2519164

[B3] Meijering E van Cappellen G Quantitative biological image analysis 2007 Imaging Cellular and Molecular Biological Function, Springer Berlin

[B4] Lamprecht M Sabatini D Carpenter A CellProfiler: free, versatile software for automated biological image analysis Biotechniques 2007 42 71 75 10.2144/000112257 17269487

[B5] Gordon A Colman-Lerner A Chin TE Benjamin KR Yu RC Brent R Single-cell quantification of molecules and rates using open-source microscope-based cytometry Nat Methods 2007 4 2 175 181 10.1038/nmeth1008 17237792

[B6] Abramoff M Magalhaes P Ram S Image processing with ImageJ Biophotonics International 2004 11 36 42

[B7] Pau G Fuchs F Sklyar O Boutros M Huber W EBImage - an R package for image processing with applications to cellular phenotypes Bioinformatics 2010 26 7 979 981 10.1093/bioinformatics/btq046 20338898 PMC2844988

[B8] Hattne J Fange D Elf J Stochastic reaction-diffusion simulation with MesoRD Bioinformatics 2005 21 12 2923 2924 10.1093/bioinformatics/bti431 15817692

[B9] Ander M Beltrao P Di Ventura B Ferkinghoff-Borg J Foglierini M Kaplan A Lemerle C Tomás-Oliveira I Serrano L SmartCell, a framework to simulate cellular processes that combines stochastic approximation with diffusion and localisation: analysis of simple networks Systems biology 2004 1 129 138 10.1049/sb:20045017 17052123

[B10] Andrews SS Addy NJ Brent R Arkin AP Detailed simulations of cell biology with Smoldyn 2.1 PLoS computational biology 2010 6 3 e1000705+ 20300644 10.1371/journal.pcbi.1000705 PMC2837389

[B11] Stiles JR Bartol TM Salpeter MM Salpeter EE Sejnowski TJ Synaptic Variability: New Insights from Reconstructions and Monte Carlo Simulations with MCell Synapses 2001 1 681 731

[B12] Tolle DP Le Novère N Meredys, a multi-compartment reaction-diffusion simulator using multistate realistic molecular complexes BMC systems biology 2010 4 20233406 10.1186/1752-0509-4-24 PMC2848630

[B13] Hamilton N Quantification and its applications in fluorescent microscopy imaging Traffic 2009 10 8 951 961 10.1111/j.1600-0854.2009.00938.x 19500318

[B14] Wollman R Stuurman N High throughput microscopy: from raw images to discoveries J Cell Sci 2007 120 Pt 21 3715 3722 17959627 10.1242/jcs.013623

[B15] Klein R Concrete and Abstract Voronoi Diagrams, Volume 200 of Lecture Notes in Computer Science. Springer-Verlag 1989 [ISBN 3540520554]

[B16] Stoma S Lucas M Chopard J Schaedel M Traas J Godin C Flux-Based Transport Enhancement as a Plausible Unifying Mechanism for Auxin Transport in Meristem Development PLoS Comput Biol 2008 4 10 e1000207+ 18974825 10.1371/journal.pcbi.1000207 PMC2565506

[B17] Mishev ID Finite volume methods on Voronoi meshes Numer Methods Partial Differential Eq 1998 14 2 193 212 10.1002/(SICI)1098-2426(199803)14:2<193::AID-NUM4>3.0.CO;2-J

[B18] Jones E Oliphant T Peterson P SciPy: Open source scientific tools for Python 2001 http://www.scipy.org/Citing_SciPy

[B19] Maeder CI Hink MA Kinkhabwala A Mayr R Bastiaens PIH Knop M Spatial regulation of Fus3 MAP kinase activity through a reaction-diffusion mechanism in yeast pheromone signalling Nature Cell Biology 2007 9 1319 1326 10.1038/ncb1652 17952059

[B20] Iverson KE A Programming Language John Wiley & Sons http://www.worldcat.org/isbn/0471430145

[B21] Knuth DE Two notes on notation Am Math Monthly 1992 99 5 403 422 10.2307/2325085

