# Supplementary material for: STSE: Spatio-Temporal Simulation Environment Dedicated to Biology
Source: BMC Bioinformatics. 2011 Apr 28;12:126. doi: 10.1186/1471-2105-12-126 (PMC3114743; doi:10.1186/1471-2105-12-126)
Supplement: Additional file 1 — Detailed description of a use case, including all individual steps of the STSE workflow with examples as well as comparative studies with state-of-the-art tools. [file 1471-2105-12-126-S1.PDF]

Additional file

In the following, we give an introduction to STSE operation and to demonstrate how to use STSE most efficiently to analyze and simulate biological systems.

In Part I, a typical STSE workflow including the modules for digitization, representation, analysis and modeling is presented in detail using the running example of an intercellular, mitogen-activated protein kinase gradient formation in mating yeast[9].

In Part II, we compare STSE modules with a selection of other available software tools which allow to perform each of the workflow substeps separately (i.e. digitization, representation, analysis or modeling).

Part III gives helpful information on STSE testing and installation procedures as well as the specification of how to reproduce the here presented analysis (i.e. how to run STSE, where to find example source codes and test data sets used in this text).

Part IV contains the STSE GUI application screenshots.

We would like to stress that we focus more on the software capabilities and its usage scenario than on the biological results. To simplify the analysis and to facilitate the usage of examples in a confirmatory way, we work on test data, inspired by the experiments and explanations presented by Maeder *et al.* in [9]. This is only an introductory exsample of how to make use of STSE, exemplified by one application area. For further examples as well as video tutorials please visit the website of STSE: `http://www.stse-software.org/`

Part I. Example case: diffusion in budding yeast

1 Biological background

Throughout this tutorial we will use the example of the intensively studied yeast pheromone MAP kinase cascade and we focus in particular on the distribution of the double-phosphorylated Fus3 ($\mathrm{Fus3}^{\mathrm{PP}}$) in a shmooing yeast cell [9]. Fus3 is part of the yeast mating pheromone signaling: upon stimulation with the pheromone α-factor, a G-protein coupled receptor is activated which leads via some intermediate steps to the recruitment of Fus3 to the forming shmoo tip.

Fus3 gets double-phosphorylated via the MAP kinase Ste7. The Fus3^{PP} gets released at the shmoo tip and can diffuse throughout the cell, which results in an observable Fus3^{PP} gradient. When reaching the nucleus Fus3^{PP}, is actively transported across the nuclear membrane and regulates transcription factors that modulate mating-specific gene expression.

To present how STSE can be used effectively we demonstrate its usage by means of analyzing and characterizing selected aspects of the Fus3^{PP} gradient in a shmooing yeast cell. For this purpose we demonstrate how to:

- Quantify the ratio of the average cytoplasm/nucleus expression of Fus3^{PP} based on fluorescence signal intensity acquired from microscopy images,
- Show gradient curves for Fus3^{PP} i) along the x-axis of the cell data image ii) around the nucleus,
- Simulate the process of the Fus3^{PP} diffusion in the cytoplasm to determine the underlying conditions that lead to the quantitative values captured in the image.

We evaluate the results of the simulations and discuss i) whether the appearance of a Fus3^{PP} gradient throughout the cell can be explained by simple diffusion and ii) how to define which conditions and model parameters are the most plausible and allow to reproduce the experimental observations with the smallest error.

1.1 Digitization

By the process of digitization we mean the generation of a digital data structure, allowing for efficient analysis, representation and modeling. The classical approach is to decompose the microscopy image into physiologically distinguishable compartments (e.g. nucleus, cytoplasm, etc.), which is called image segmentation [14, 3]. Usually image segmentation results in a data structure linking the compartments with pixels. STSE differs from this approach by introducing an abstract, intermediate layer composed of so-called subcompartments. To generate this layer each compartment is divided into subcompartments which have the geometry of polygons and are organized in such a way that they fill the entire compartment and do not overlap with each other. The default geometry is automatically composed of equilateral hexagons. The purpose of introducing this abstract layer is to allow for adjusting the digitization precision separately for different compartments, which is useful when it comes to analysis and modeling.

In the running example we focus on the Fus3^{PP} gradient in the cytoplasm. In this case it is useful to keep high precision in the cytoplasm compartment in order to capture the gradient. Since our analysis includes hypothesis about the Fus3^{PP} distribution neither outside the cell nor in the nucleus (motivation for this is presented in 1.3), we may use varying "subcompartment densities" in these compartments as presented in Figure 1. The user is able to fine-tune the geometry of subcompartments using the GUI editor to match different analysis

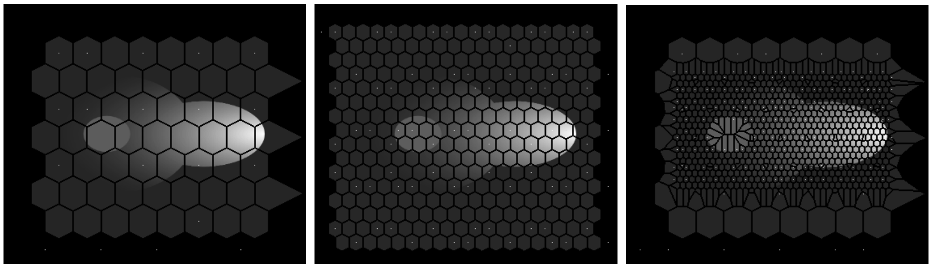

(a) (b) (c)

Fig. 1: Different "subcompartment density" variants: (a) rough regular digitization (b) more refined, regular digitization (c) refined, irregular representation edited with the STSE GUI.

and modeling requirements (Figure 1c). Without using the abstract subcompartment layer, the "subcompartment density" would have to remain constant and would be defined by pixels.

Another task related to the digitization of image data is the acquisition of subcompartment types (i.e. determining for each abstract subcompartment its affiliation to a cellular compartment). This task can be performed via the GUI or a Python script. Although a subcompartment type can be set manually, in both cases the recommended way is to use an automatic protocol based on binary masks. These binary masks are based on original microscopy images and can be prepared with 3rd party segmentation algorithms (e.g. implemented in ImageJ). The choice of the segmentation algorithm depends highly on the particular problem and there already exist a multitude of advanced software packages dedicated to this task. Therefore in the current version of the STSE we decided to use already segmented images as the starting point of the proposed workflow and leave the choice of the optimal segmentation method to the user. Each subcompartment is associated with only one compartment type. When a conflict occures (e.g. in the case of overlapping binary masks) the user can influence subcompartment types by changing the order of application of the binary masks or by defining subcompartment types manually.

In the Fus3 example we use binary masks for localization of the following cell types (Figure 2): the cytoplasm (`shmoo_00.png`), the nucleus (`shmoo_01.png`), the cell membrane (`shmoo_02.png`), the nuclear membrane (`shmoo_03.png`), the shmoo tip (`shmoo_04.png`).

In the running Fus3 example these mask files are used to acquire the subcompartment types by either GUI or a Python script. Both methods are covered in subsections 1.1.1 and 1.1.2, respectively. The results of such an operation are presented in Figure 3.

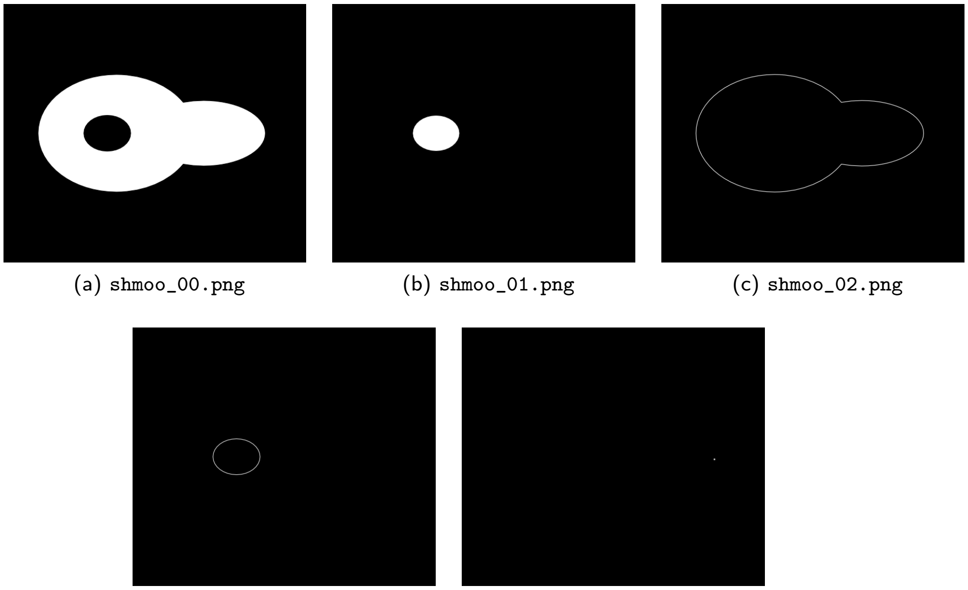

(a) shmoo_00.png (b) shmoo_01.png (c) shmoo_02.png

(d) shmoo_03.png (e) shmoo_04.png

Fig. 2: Binary masks created from raw data images. The images present the binary masks for (a) the cytoplasm, (b) the nucleus, (c) the cell membrane, (d) the nuclear membrane and (e) the shmoo tip (a single pixel is sufficient to mark the shmoo tip).

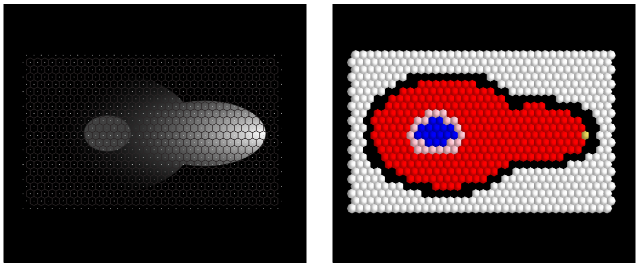

(a) (b)

Fig. 3: Subcompartment types assignment: (a) mesh showing the geometry of subcompartments (b) types of subcompartments acquired from the binary masks. Different sphere colors depict different compartment identities: white - outside, black - the cell membrane, blue - the nucleus, red - the cytoplasm, pink - the nuclear membrane, yellow - the shmoo tip.

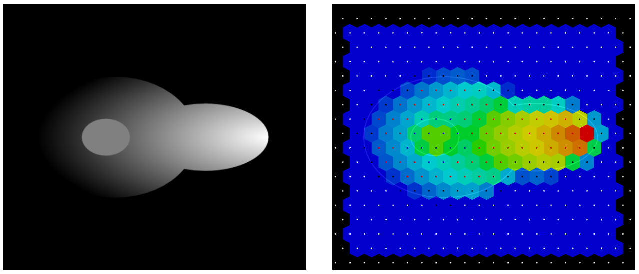

(a) (b)

Fig. 4: Signal quantification (a) indexed color image of Fus3^{PP} localization (`shmoo_05.png`) (b) result of Fus3^{PP} signal quantification based on the indexed color image. Small spheres depict the compartment types (as in the Figure 3).

The automatic acquisition of the signal from the microscopy image is another demanding task and provides the basis for the subsequent analysis and modeling steps. For this purpose we use indexed color images (e.g. standard light/confocal microscopy images) corresponding to molecular concentrations of molecules of interest. In the running Fus3 example this image resulted from fluorescence lifetime imaging microscopy (FLIM) experiment which determines the intracellular localization of Fus3^{PP} (`shmoo_05.png`). For the sake of simplicity we use a test data image inspired by the experiments described in [9], in which the activated form of Fus3 has been directly detected. Again, this task can be performed either via the GUI or with a Python script. Both methods are covered in subsection 1.1.1 and 1.1.2, respectively. The result is presented in Figure 4.

To summarize, the inputs for the digitization procedure are i) binary masks ii) indexed color images. The outcome of the digitization step is a manageable amount of subcompartments covering the microscopic image. Each subcompartment is allocated with a specific compartment type and the average intensity of protein(s) of interest acquired from input data.

1.1.1 GUI

The complete procedure for performing the steps presented in Section 1.1 with the GUI application STSE Compartment Editor is presented in Algorithm 1 (images presenting these actions are shown in the Part IV of this document; additionally, video tutorials covering this subject are provided on the webpage of the STSE project).

Algorithm 1 Digitization procedure in STSE.

1. Load indexed color image with fluorescent protein of interest by using the button "Load background" (Figure 11),
2. Add Voronoi centers by choosing Actions -> Add Voronoi centers in the menu bar. Then define in the Actions menu the properties of the Voronoi centers and execute the task (Figure 12). Now, if it is desired, it is possible to edit the mesh by dragging the Voronoi centers (check help tab in the GUI application for editing related keystrokes and mouse actions),
3. Calculate expression by choosing Actions -> Calculate expression in the menu bar. Then define your cell property name and execute the task (Figure 13); the chosen property name must be one of the properties visible in the Selected center tab,
4. Go to the Visualization menu and click "Display cell property" and choose the cell property to be displayed. The averaged pixel intensity in each polygon is represented by a color scheme.
5. To acquire the subcompartment types:
   (a) Hide the surface of the mesh, to see the binary masks loaded in the next step. This is done via the button "View the Mayavi pipeline" on the left. Go to Mayavi Scene 1 -> VTK Data -> Colors and legends -> surface . Right click on surface and press "Hide/Show". The surface, including also the colors from step 4, is hidden now.
   (b) Load binary image for cellular structure of interest, e.g. cytoplasm. This can be done via the button "View the Mayavi pipeline" on the left. Go to Mayavi Scene 1 and then to the image which was used for the background (here the indexed color image). Via the timestep bar on the right one can scroll through the images, if they are named properly.
   (c) Define cell type for the structure chosen in the previous step, e.g. the cytoplasm. In the left part of the main window the picture with the white cytoplasm and black background is shown. Go to the menu bar and choose Actions -> Define cell types. Then go to the Actions menu on the right and define the cell type. In this case you name it "B" (by default subvalues are labeled with "A"; the next defined cell type would be "B", then "C" and so on) and press execute (Figure 14). All subcompartments which contain white pixels will now acquire the assignment "B". It is possible to change these default names by subclassing the GUI application (this is beyond the scope of this document).
   (d) Repeat steps 5b and 5c for all other cellular structures of interests,
   (e) Display the cell property of interest together with cellular structures: Go again to Mayavi Scene 1 -> VTK Data -> Colors and legends -> surface, right click surface and press "Hide/Show". The surface including the colors representing the cell property will be displayed again. With a left click on "Surface" and selecting "Actor" on the right one can make modifications like changing the opacity of the surface. This option is convenient in our case since via reducing the opacity of the surface one can overlay the surface with the binary masks to get a better impression of the localization of the subcompartments (Figure 15).

1.1.2 Script

Since STSE allows for Python scripting, it is possible to perform all the steps described in the previous section by using a Python script.

The script `10_04_20_create_shmoo_data.py`[1] shows how to automatise the GUI operations performed with the STSE Compartment Editor.

1.2 Representation and analysis

The digitized tissue is kept in a design called "WalledTissue2D", being an internal STSE data structure. This planar 2D grid structure involves less constraints than the Voronoi 2D mesh used for defining the mesh geometry with the GUI (described in Section 1.1) and thus allows for more latitude in defining polygonal geometries (e.g. including non-convex ones). It is also possible to keep, access and edit the physiological information related to subcompartments. This information can be used to generate different views of the studied object (e.g. values such as morphogen concentrations, numbers of neighbours, subcompartment areas). These manipulations are realized via Python scripts and may be used to inspect different properties of the representations.

The examplary analysis of Fus3^{PP} is presented in `10_06_17_analysis_shmoo.py`. This file demonstrates common tasks performed with STSE such as inspecting or editing:

- geometrical properties of the subcompartments/compartments,
- physiological properties of the subcompartments/compartments,
- topological properties of the subcompartments,
- mesh (removing or resizing the subcompartments),

to name just a few.

For the purpose of the Fus3^{PP} gradient example analysis we learn the following (`10_06_17_analysis_shmoo.py`):

- the distribution of Fus3^{PP} in the cytoplasm along the x-axis in a central part of the analyzed cell is exponential (Figure 5c),
- the distribution of Fus3^{PP} around the nucleus reaches its maximum in the point closest to the shmoo tip (Figure 5d),
- the average Fus3^{PP} signal in the nucleus is 64.0 (which is $\approx 25\%$ of max signal measured in the image),
- the average Fus3^{PP} signal in the cytoplasm is 52.07 (which is $\approx 20\%$ of max signal measured in the image),

[1] to check how to access all files used in this text (and printed with `Typewriter` font) please consult Part III of this text.

- the ratio of averaged Fus3^{PP} signal in the cytoplasm to nucleus is equal to 0.81.

In Part II, these data are compared with values computed with ImageJ.

1.3 Modeling

In the following we use the previously acquired quantified and structured data to create a dynamic model of Fus3^{PP} diffusion. According to the STSE dataflow paradigm, the mechanistic model of the studied process needs to be formalized. This is done by first making fundamental statements using natural language (e.g. by experts in biology) and converting them into a set of ODE equations. These steps allow for a formal description of the interplay of different actors (like chemical molecules) and the different cellular compartments with specified kinetic rules on diffusion, chemical reactions, transport, etc. This formal description creates a spatial complex dynamic system. The understanding of such a system profits significantly from the use of computational techniques (e.g. simulations), since they allow us to to reproduce the system's kinetics.

For the example of Fus3^{PP}, diffusion the following mechanism is proposed by biologists [9]:

- Fus3 gets double-phosphorylated by Ste7 at the scaffolded MAPK cascade now located at the shmoo tip and is released to the cytoplasm,
- Fus3^{PP} is diffusing freely in the cytoplasm,
- Fus3^{PP} gets dephosphorylated while diffusing,
- Fus3^{PP} requires active transport to enter the nucleus.

As in the previous sections, we focus on the properties of the Fus3^{PP} gradient. Therefore we exclude processes such as i) mechanisms of the stimulation of Fus3 (i.e. signaling via Ste7) and ii) different mechanisms allowing Fus3^{PP} to enter the nucleus. The formulation of the kinetic model of Fus3^{PP} is now defined as follows:

- Fus3^{PP} is appearing in the shmoo tip compartment,
- Fus3^{PP} is diffusing freely in the cytoplasm compartment,
- Fus3^{PP} gets dephosphorylated while diffusing,
- Fus3^{PP} is unable to cross the cellular / nuclear membrane compartments.

With this model we are able to verify whether it is possible to reproduce the qualitative properties of the Fus3^{PP} gradient observed in the digitized images. For this purpose the model is translated into the following system of differential

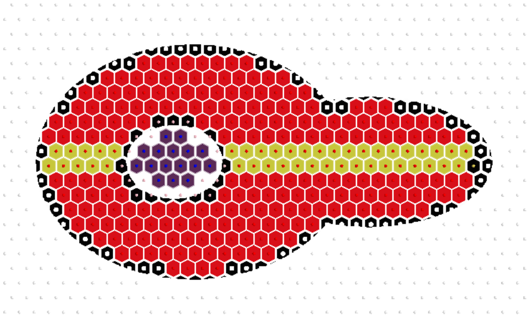

(a)

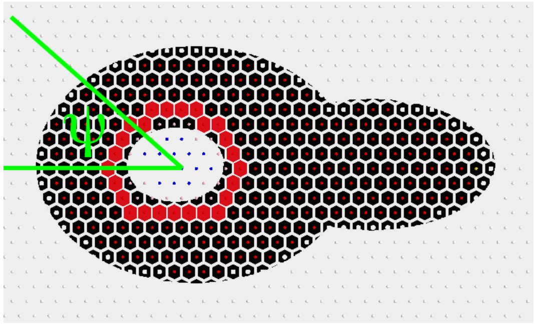

(b)

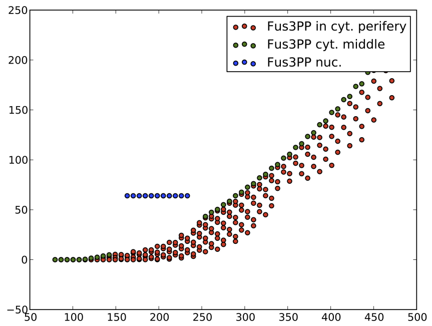

(c)

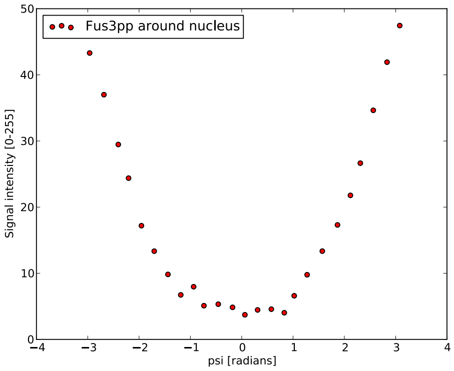

(d)

Fig. 5: Fus3^{PP} profiles along the x-axis and around the nucleus. (a) subcompartment locations used to distinguish the curves in (c); (b) subcompartment locations and ψ definition used in (d); (c) Fus3^{PP} profiles along the x-axis; (d) Fus3^{PP} profiles around the nucleus.

equations (for each subcompartment i we will have one equation describing the changes of Fus3^{PP} concentration)[2]:

$$\begin{aligned}\frac{\partial FUS3^{PP}{}_i}{\partial t} = & -\sum_{n\in N_i} \frac{S_{i\to n}}{V_i}\gamma_{FUS3^{PP}}\left(FUS3^{PP}{}_i - FUS3^{PP}{}_n\right)_{i\to n}[n\in A,B]+ \\ & +\alpha_{FUS3^{PP}}\,[i\in B] - \beta_{FUS3^{PP}} FUS3^{PP}{}_i\end{aligned}$$

where:

- $FUS3^{PP}{}_i$ is the concentration of Fus3^{PP} in the subcompartment i,
- $\gamma_{FUS3^{PP}}$ is the diffusion constant for Fus3^{PP},
- $\alpha_{FUS3^{PP}}$ is the rate of Fus3^{PP} release in the shmoo tip,
- $\beta_{FUS3^{PP}}$ is the rate constant of Fus3^{PP} dephosphorylation,
- $S_{i\to n}$ is the area of contact surface between subcompartments i and n,
- V_i is the volume of subcompartment i,
- $i \in A$ / $i \in B$ if i belongs to cytoplasm (A) or shmoo tip compartment (B),
- N_i is a set of neighbour subcompartments for subcompartment i,
- $[\psi] = \begin{cases} 1 & \text{if } \psi \text{ is True} \\ 0 & \text{otherwise} \end{cases}$, (e.g. $[n \in A \cup B]$ evaluates to 1 when n is element of A or B) [6, 7].

To complete the model it is required to define the parameters $(\alpha_{Fus3}, \beta_{Fus3}, \gamma_{Fus3})$ and the initial conditions. All values can be estimated from the literature or chosen arbitrarily. Additionally, initial conditions can be acquired from the digitization step of the image data. Since we focus on the illustration of the software functioning, for the sake of simplicity in the running example we use arbitrary parameter sets and initial conditions.

The ODE system then becomes encoded by the modeler in Python language. At this stage STSE does not provide automatic support for such an operation. The examples available with this document and with STSE sources show an exemplification and introduce the methodology and good coding practice.

For the running example an implementation of the Fus3^{PP} model is provided in `10_06_22_diffusion_shmoo.py`.

Here it is assumed that $(\alpha_{Fus3}, \beta_{Fus3}, \gamma_{Fus3}) = (0.1, 0.1, 100)$ and the initial concentration of Fus3^{PP} in every subcompartment is equal to 0. An animation showing the kinetics of Fus3^{PP} distribution obtained with the implemented model is available as additional file `2.avi`.

[2] Before studying the equation below it is suggested to check for the definition of function $[] : \psi \to \{0, 1\}$.

Simulations in STSE can be used e.g. to estimate the values of Fus3^{PP} model parameters based on the image data. The scenario of such a study could look as follows (this is an adaptation of a common technique called parameter fitting):

1. Prepare a set of different tuples of parameters:

$$S = \{(\alpha_{Fus3}, \beta_{Fus3}, \gamma_{Fus3}), (\alpha_{Fus3}, \beta_{Fus3}, \gamma_{Fus3})', ...\}$$

2. For every element $s \in S$ run the simulation to obtain the steady state. Let F_s describe the distribution of Fus3^{PP} for parameter set s in the steady state.

3. Lets denote $\bar{F_s}$ the normalized distribution of F_s and $\bar{F}$ the normalized distribution of Fus3^{PP} obtained from the original image (normalization means scaling of the values for Fus3^{PP} so they belong to the segment $[0, 1]$).

4. The most plausible set of parameters would be the one minimizing the difference $\left|\bar{F_s} - \bar{F}\right|$

For example the steady state concentrations $F_{(0.01,0.001,100)}$ and $F_{(0.01,0.001,50)}$ for two different simulations are presented in Figure 6. An animation showing the kinetics of Fus3^{PP} evolution for the steady state $F_{(0.01,0.001,100)}$ is shown in additional file `3.avi`. This simulation differs by changing the model property γ_{FUS3} from 50 to 100. For further illustration Figure 7 presents the difference between $\bar{F}_{(0.01,0.001,50)}$ and $\bar{F}$ (generated with `10_07_01_compare_fus3_distributions.py`). As mentioned in the algorithm description above this difference can be used to discriminate between parameter sets.

The running example model yields the highest error in the nuclear compartment. This is consistent with our expectations: the test data set images suggest that Fus3^{PP} is present in the nuclear compartment (Figure 4), but in the specification of the model we skipped the mechanism of Fus3^{PP} transport via membranes, which results in the Fus3^{PP} concentration in the nuclear compartment being equal to 0. Therefore for any parameter set the concentration of Fus3^{PP} in the nuclear compartment will be different from the test data set. To correct this property, the model should be extended by an assumption of Fus3^{PP} transport via the nuclear membrane.

Part II. Comparison of STSE with other tools

2 Introduction

A variety of image processing-, analysis- or modeling-packages already exist, either commercial or open source. In the following we compare the STSE modules

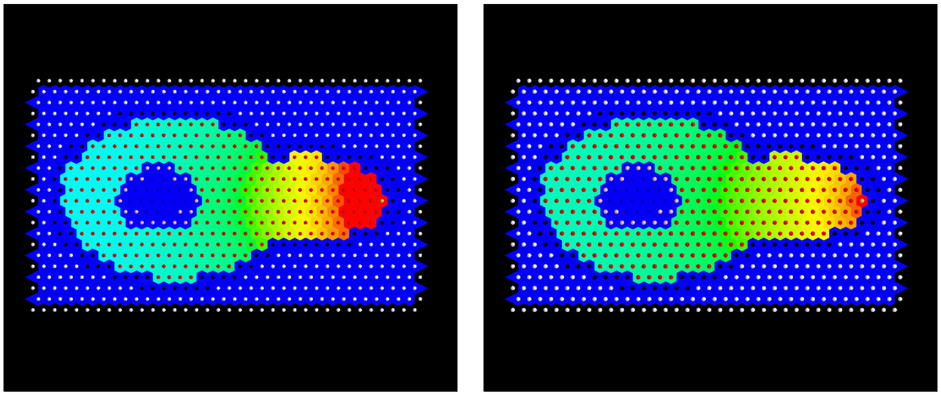

(a) (b)

Fig. 6: Steady state distributions of Fus3^{PP} for two different parameter sets $(\alpha_{Fus3}, \beta_{Fus3}, \gamma_{Fus3})$: (a) $(0.1, 0.1, 100)$, (b) $(0.1, 0.1, 50)$. We observe that the gradients have different slopes, which is due to the difference in the diffusion constant γ_{Fus3}. To visualize Fus3^{PP} concentrations, a colormap is used where blue depicts low values and red depicts high values.

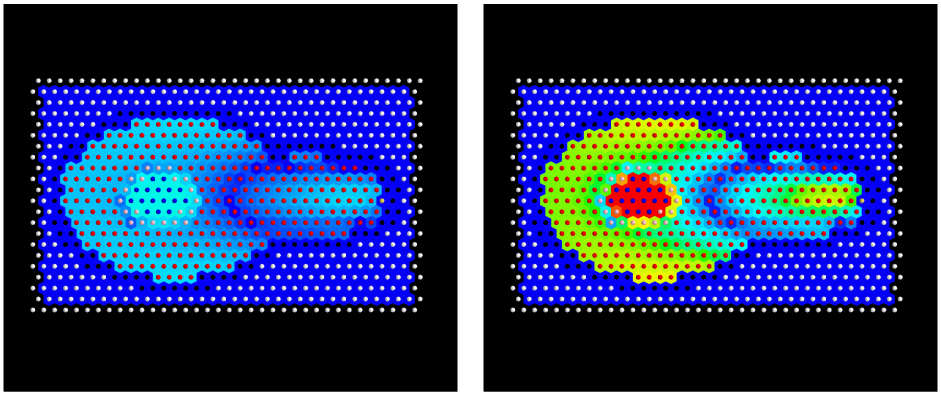

(a) (b)

Fig. 7: Difference of Fus3^{PP} concentration between $\bar{F}_{(0.01,0.001,50)}$ and $\bar{F}$ (which approximates the error). (a) shows an $E = \left|\bar{F}_{(0.01,0.001,50)} - \bar{F}\right|$ (the overall error did not exceed 20% percent) (b) shows the $E/max(E)$ (when 100% of error was observed in the center; it is important to note, that the model did not allow the Fus3^{PP} to enter the nucleus compartment). To visualize E, a colormap is used where blue depicts low values and red high ones.

to selected tools: ImageJ [1] and MesoRD [4]. The first steps of the STSE workflow (digitization, representation and analysis) are demonstrated in comparison with ImageJ, and the modeling module is compared to the MesoRD.

3 Digitization

By the process of digitization we mean the generation of a digital data structure, allowing for efficient analysis, representation and modeling. The classical approach is to decompose the microscopy image into physiologically distinguishable compartments (e.g. nucleus, cytoplasm, etc.) which is called image segmentation [3, 14, 8]. Various algorithms for segmentation already exist and the choice of the optimal algorithm depends on the structure of the image and of the objects of the interest.

In STSE there is no segmentation algorithm implemented. The user needs to provide binary masks created with 3rd party software, such as e.g. ImageJ. The preparation of binary masks is beyond the scope of this text.

4 Representation and analysis

In this section we compare STSE with ImageJ in the procedures for image representation and analysis. Once again we exemplarily use the distribution of $Fus3^{PP}$. The procedure of preparing the data in STSE is covered in Section 1.1 and the actual analysis is presented in Section 1.2.

4.1 Task: Calculate the cytoplasm over nucleus ratio of the $Fus3^{PP}$ signal in yeast

4.1.1 ImageJ

In ImageJ the procedure would be to determine the average concentration of $Fus3^{PP}$ in the cytoplasm as well as in the nucleus and then to calculate the ratio manually. The average $Fus3^{PP}$ signal in the nucleus is 64.0, and the average $Fus3^{PP}$ signal in the cytoplasm is 51.27, though the ratio between these values being equal 0.801. A detailed description of the procedure with ImageJ is presented in Algorithm 2.

4.1.2 STSE

To calculate the cytoplasm over nucleus ratio in STSE we use a Python script which automatically performs the digitization step described in 1.1. As it has been already summarized for STSE, the average $Fus3^{PP}$ signal in the cytoplasm is 52.07, the average $Fus3^{PP}$ signal in the nucleus is 64.0, and so the ratio between these values is equal to 0.81.

Algorithm 2 ImageJ procedure to calculate the cytoplasm over nucleus ratio of the $\mathrm{Fus3}^{\mathrm{PP}}$ signal

1. Load the binary masks for the cell membrane and for the nucleus into ImageJ. The images can be seen in Figure 2c (shmoo_02.png) and 2b (shmoo_01.png).

2. Use the wand (tracing) tool to select the cell membrane as well as the nuclear membrane and store both selections in the ROI manager.

3. Determine the concentration of the protein in the cytoplasm via:

   (a) Go to the image of the cell membrane (Fig. 2c), choose the selection for the cell membrane from the ROI manager and press Edit -> clear outside in the menu bar.

   (b) Go to the image of the cell membrane (Fig. 2c), choose the selection for the nucleus from the ROI manager and press Edit -> clear in the menu bar.

   (c) Steps 3a and 3b result in picture 8a.

   (d) Load the indexed color image from Fig. 4a (shmoo_05.png) into ImageJ and combine it with Fig. 8a by using Process -> ImageCalculator. Choose Operation: "Max" and define Image1 and Image2 as Fig. 4a and Fig. 8a, respectively. The result can be seen in Fig. 8b.

   (e) The measurement will be performed by using a threshold. Therefore go to Analyze -> Set Measurement and check "Limit to Threshold".

   (f) Change the image type to 8-bit via Image -> Type set to 8-bit.

   (g) Go to Image->Adjust->Threshold. Set the lower threshold to 0 and the upper threshold to 254, which means that every pixel of the image will be included into the measurement, which is not completely white and therefore not completely saturated.

   (h) Analyze -> Measure calculates the average pixel intensity in the cytoplasm. The cytoplasm has an area of 62640 pixels with 51.27 average pixel intensity.

4. Determine concentration of the protein in the nucleus via:

   (a) Use the selection of the nuclear membrane from step 2 and load it into picture 4a.

   (b) Analyze->Measure calculates the average pixel intensity in the nucleus. The nucleus has an area of 4598 pixels with an average mean intensity of 64.0 .

5. Manual calculation of the ratio between of the cytoplasmic and nuclear concentrations results in 0.801.

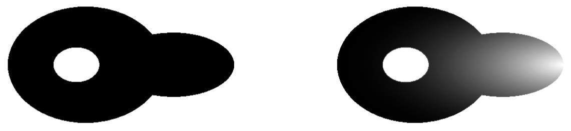

(a) (b)

Fig. 8: ImageJ workflow images. (a) Binary mask for the cytoplasm (b) distribution of $\mathrm{Fus3}^{\mathrm{PP}}$ inside the cytoplasm.

4.1.3 Comparison

Both tools allow us to compute the ratio between the $Fus3^{PP}$ signal in the cytoplasm and the nucleus. The value obtained by ImageJ is *exact*, since ImageJ operates on pixels. STSE provides an *approximation*, since it uses abstract subcompartments to group pixels (e.g. as a consequence some pixels belonging to nuclear compartment inputed their value to cytoplasm subcompartments). The relative approximation error in our example is $(0.81 - 0.801)/0.801 \approx 1\%$.

The precision is regulated in STSE by decreasing of the subcompartment size. This increase in precision induces slower execution of the modeling routines. It is a choice of modeler to prepare the grid in such a way that both, the precision and speed of modeling routines will be optimal.

STSE allows for automatisation of such a measurements (via Python). In ImageJ this requires the usage of macros, plugins and scripts (via Java-like, Java and JavaScript). Binary masks can be used for automatization in STSE. Contrary, they need to be pre-processed in ImageJ.

Due to the current implementation, it is difficult to deal with non-coherent selections in ImageJ (i.e. selections of two or more separated regions). As a consequence, writing a plugin or script to deal with the measurements of the signal in compartments which are not coherent (e.g. cells often have multiple separated mitochondria) might be currently not possible. STSE does not suffer from this effect.

4.2 Task: show a gradient curve for $Fus3^{PP}$ throughout the cell

4.2.1 ImageJ

In ImageJ this can be done by the following procedure:

1. Load the image `shmoo_05.png` into ImageJ.
2. Choose a straight line selection through the center of the cell.
3. Go `to` Analyze->Plot Profile, which will open 2 new windows showing the plot profile ands the intensities of each pixel along the line.

The image including the selection, the line profile and part of a table with the intensities of pixels along the line in table form is shown in Figure 9.

ImageJ offers also the feature of using a rectangular selection and plotingf the profile of the average pixel intensity of each vertical line along the horizontal distance of the selection. In Figure 10 we show the result of a possible rectangular selection. With this procedure one can capture some of the spatial information, but because of the rectangular shape of the selection one either misses cellular information (if the rectangle is chosen too narrow) or one includes too much background (if the rectangle is chosen too broad, as in Figure 10).

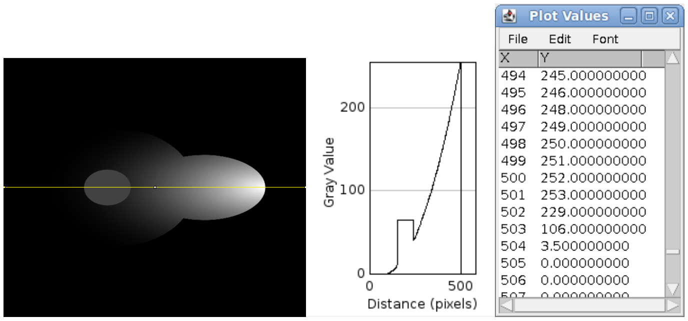

(a) (b) (c)

Fig. 9: Plot profile with straight line selection. (a) Cell with straight line selection (b) Plot profile with straight line selection (c) Part of the line profile in tabular form.

4.2.2 STSE

The profile obtained with STSE is presented in Figure 5. Since STSE is embedded in Python it allows to use many advantageous libraries. In the case of the running example Scipy is used to prepare the figures (`10_06_17_analysis_shmoo.py`). It is also possible to perform more complex analysis e.g. fitting the curves based on the measured data using Scipy routines (not presented since it is beyond the scope of this text).

4.2.3 Comparison

Both tools allow for computing the profile plot along the x-axis of the cell (with STSE approximated as green dots in Figure 5). As stated in Section 4.1.3, the value obtained by ImageJ is *exact* whereas STSE provides an *approximation*, because it uses abstract subcompartments.

Inspecting of Figure 9b and Figure 5 shows that the exponential decrease of Fus3^{PP}along the cell center as well as the increase in the nucleus can be captured reasonably well with both tools. Nevertheless, if one is interested in the distribution of Fus3^{PP} over the whole cell, there is no constitutive way to do so in ImageJ (however plugins allow to perform similar actions). The illustrated example of using a rectangular selection shows that this method is suitable only for rectangular cells. If the cellular shape is not, by chance, rectangular, the measurement error is high. Furthermore, in STSE it is possible to plot various profiles for any selected subcompartments.

In ImageJ basic analysis is performed via the GUI, but an extensive analysis again requires the usage of Macros, Plugins or Scripts (via Java-like, Java or

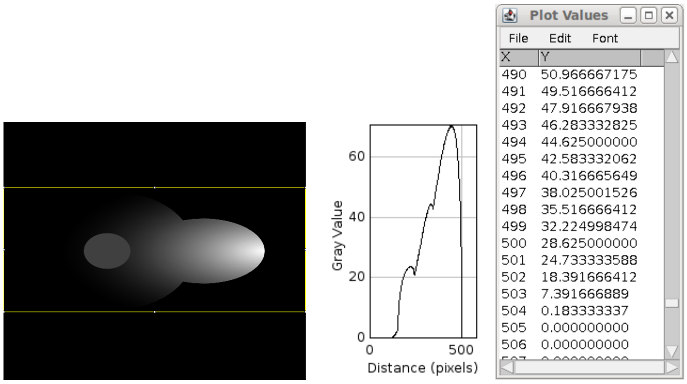

(a) (b) (c)

Fig. 10: Plot profile with rectangular selection. (a) Cell with rectangular selection (b) plot profile with rectangular selection (c) part of the rectangular profile in tabular form.

JavaScript). STSE requires using Python in both basic and extensive analysis. In the latter case the automatisation of tasks via Python allows for faster implementation.

Additionally, due to the current implementation of ImageJ, it is difficult to deal with non-coherent selections in ImageJ (i.e. selections of two or more separated regions). As a consequence, writing as Plugin / Script to deal with the measurements of the signal in compartments which are not coherent (e.g. cells often have multiple separated mitochondria) might be currently impossible. STSE does not suffer for this effect.

5 Modeling

Over the last years numerous modeling tools were developed e.g. MesoRD, Matlab, MCell or VCell to name just a few of them (for a short review please see [10]). These software packages differ significantly in terms of the algorithms used or the specific purpose they were designed for. To keep the presentation of the STSE package simple we decided to chose MesoRD [4] as a reference since corresponds the best to the STSE software. In the following we list explicitly the differences between the modeling strategies of both software packages.

5.1 2D versus 3D modeling

One of the main differences between MesoRD and STSE is that subcompartments in MesoRD are 3D entities (i.e. volumes and therefore subvolumes) whereas subcompartments in STSE are 2D entities (i.e. surfaces). However it is possible to use STSE subcompartments as abstract 3D entities in the simu-

lations (as presented in the running example in 1.3). The main reasons for this design choice in STSE are:

1. The GUI editing, representing or inspecting of 2D meshes is simpler than for 3D meshes. One of the goals of STSE is to allow the user to perform the GUI mesh editing operations, therefore we did not want to clutter the interface.

2. STSE is aimed to work also as an abstract tool for "sketching" the dynamics of spatial complex systems, without any real microscopic images (this is not covered in this document). In this mode it is useful to prepare the meshes as well as to perform spatial simulations. To keep these procedures simple the 2D approach is in our opinion a better design choice.

3. In terms of quantitative modeling 2D data preprocessing is far less complex than dealing with 3D stacks (e.g. the non-linear, medium dependent, signal attenuation in the z-axis of the 3D stack, data structures and algorithms required to analyze 3D data, size of data). On the other hand, STSE is well suited to perform the spatial simulations in a single 3D layer, which solves the preprocessing issues. Additionally, modeling of a single 3D layer is useful in many biological problems (e.g. [11, 12]), therefore in our opinion STSE is still useful despite its 3D limitations.

4. Finally it is the choice of the modeler to implicitly model 3D with 2D (e.g. diffusion constants can be recalculated, processes can be approximated). It is demonstrated in the running example.

2D datastructure allow us to use microscopic image data to compare with the simulation results at different time points, which is a major advantage of STSE workflow. This is not possible in MesoRD.

5.2 Simulation strategy

For simulations MesoRD uses an algorithm called the Next Subvolume Method (NSM) to simulate the model stochastically [2], whereas STSE is currently based on Ordinary Differential Equations (ODEs) and simulates the model deterministically. It is important to note, that STSE is not imposing any solver strategy on the user. In fact, it provides only the datastructure API and examples how to perform spatial simulations with LSODE library solvers [5]. Therefore the choice of simulation algorithm and its implementation depends on the user. It is possible to use stochastic algorithms similar to the NSM within the STSE environment. Unfortunately, there are currently neither examples nor tutorials how to prepare such a simulation.

5.3 Compartment geometry

In MesoRD geometry is defined with MesoRD extension to Systems Biology Markup Language SBML standard. It is based on the constructive solid geometry format (CSG) [13], by applying combination operations on simple 3D

objects. In STSE, one gets the compartment geometry based on the microscopy images by using binary masks or specifies it with the GUI.

5.4 Subcompartment geometry

In MesoRD subcompartments must be cubes of the same size. STSE allows to use default equal size, hexagonal subcompartments. Later, the hexagonal lattice can be altered via GUI or Python script to adjust individual subcompartment properties like size and shape. While using GUI each subcompartment shape is a 2D convex polygon.

5.5 Initial conditions

The major advantage of STSE in comparison to MesoRD is, that the output of the digitization process can be directly used as an input or comparison for the modeling process. In MesoRD the user has to define the initial concentrations for each cellular compartment with information available in the literature. STSE has the major benefit that on the one hand, data can be directly used from previously imported data images and on the other hand, concentrations for even individual subcompartment can be defined with a Python script. Therefore it is possible to start the simulation with a gradient in one cellular compartment, which is not possible in MesoRD. It is described in detail in section 1.3.

5.6 Kinetics specification

The input for the kinetic modeling in MesoRD is based on the SBML, although with some minor changes to make it applicable for simulations in space. In STSE the kinetics of the system needs to be specified by the implementation in Python. In MesoRD as well as in STSE one has to define the kinetics for diffusion and chemical reactions. While MesoRD just includes chemical reactions and diffusion, in STSE also further mechanisms like special rules or directed transport are possible (the modeler has the freedom to define the Python code to specify any dynamics).

Part III. Installation issues and SBOS

6 Dependencies

STSE is freely available to the research community from `http://www.stse-software.org/` Since the software uses third party components, its functioning depends on the availability of the following libraries and applications for the given platform:

- Python (`http://www.Python.org/`),
- Openalea (`http://openalea.gforge.inria.fr/`),

- Mayavi2 (`http://code.enthought.com/projects/mayavi/`),
- Qhull (`http://www.qhull.org/`),
- NetworkX (`http://networkx.lanl.gov/`).

The framework is tested on Linux, but in principle it is possible to run the software on any platform in which these dependencies are combined.

7 Layout of files and data used in the text

All files used in this text are accessible via SVN from the open source community developer site: `http://sourceforge.net` The source files used in the text (e.g. `10_06_17_analysis_shmoo.py`) are placed in:

`http://stse.svn.sourceforge.net/svnroot/stse/trunk/src/stse/examples/`

The image data used in the text (e.g. `shmoo_01.png`) is placed in:

`http://stse.svn.sourceforge.net/svnroot/stse/trunk/data/10-04-27-shmoo/`

8 Running STSE from SB.OS

To test the software without labored installation procedure (described on the project website), it is recommended to use the software directly from the live DVD Linux distribution, SB.OS (`http://www.sbos.eu/`), which comes with a comprehensive list of other systems biology software. Running SB.OS is possible by booting up the computer with SB.OS DVD in the drive or via Virtual Machine (e.g. using software such as VMWare).

Inside the file system of SB.OS the source files used in the text (e.g. `10_06_17_/analysis_shmoo.py`) are placed in the folder:

`/usr/local/stse/src/stse/examples/`

The running example image data used in the text (e.g. `shmoo_01.png`) are placed in the folder:

`/usr/local/stse/data/10-04-27-shmoo/`

To test the examples used in the text:

1. After starting SB.OS it is recommended to update the STSE software before usage (a network connection is required for this operation. It ensures that the user has access to the newest version of the software and is helpful in case of reporting bugs). To perform this action one can use Menu: Applications / Systems Biology / STSE / STSE-Update.

2. To test various source codes from the text use Menu: Applications / Systems Biology / STSE / STSE-iPython[3]. Afterwards, when the console appears, one can change the directory to the folder with examples and run one of them by issuing the following commands (e.g. to run `10_06_17_analysis_shmoo.py`):

   ```
   cd /usr/local/stse/src/stse/examples/
   run 10_06_17_analysis_shmoo.py
   ```

3. To test algorithm 1 use STSE GUI application Compartment Editor from Menu: Applications / Systems Biology / STSE / STSE-CE. The example data for the application are placed in:

   `/usr/local/stse/data/10-04-27-shmoo/`

References

[1] MD Abramoff, PJ Magalhaes, and SJ. Ram. Image processing with imagej. *Biophotonics International*, 11:36–42, 2004.

[2] J. Elf and M. Ehrenberg. Spontaneous separation of bi-stable biochemical systems into spatial domains of opposite phases. *Syst Biol (Stevenage)*, 1(2):230–236, Dec 2004.

[3] Nicholas Hamilton. Quantification and its applications in fluorescent microscopy imaging. *Traffic*, 10(8):951–961, Aug 2009.

[4] Johan Hattne, David Fange, and Johan Elf. Stochastic reaction-diffusion simulation with mesord. *Bioinformatics*, 21(12):2923–2924, Jun 2005.

[5] A. C. Hindmarsh. *ODEPACK, A Systematized Collection of ODE Solvers.* IMACS Transactions on Scientific Computation. North-Holland Amsterdam, 1983.

[6] Kenneth E. Iverson. *A Programming Language.* John Wiley & Sons.

[7] Donald E. Knuth. Two notes on notation. *Am. Math. Monthly*, 99(5):403–422, May 1992.

[8] Vebjorn Ljosa and Anne E Carpenter. Introduction to the quantitative analysis of two-dimensional fluorescence microscopy images for cell-based screening. *PLoS Comput Biol*, 5(12):e1000603, Dec 2009.

[9] C I Maeder, M A Hink, A Kinkhabwala, R Mayr, P I H Bastiaens, and M Knop. Spatial regulation of fus3 map kinase activity through a reaction-diffusion mechanism in yeast pheromone signalling. *Nature Cell Biology*, 9:1319–1326, 2007.

[3] To run the examples it is recommended to use iPython shell with special flags (this is due to the threading issues in different libraries used by STSE; detailed explanation is beyond the scope of this text). To use the full capabilities of STSE Compartment Editor simply run `iPython` with the flag `-wthread`.

[10] Haluk Resat, Linda Petzold, and Michel F Pettigrew. Kinetic modeling of biological systems. *Methods Mol Biol*, 541:311–335, 2009.

[11] Richard Smith. *Simulation Models of Phyllotaxis and Morphogenesis in Plants*. PhD thesis, August 2007.

[12] Szymon Stoma, Mikael Lucas, Jérôme Chopard, Marianne Schaedel, Jan Traas, and Christophe Godin. Flux-Based Transport Enhancement as a Plausible Unifying Mechanism for Auxin Transport in Meristem Development. *PLoS Comput Biol*, 4(10):e1000207+, October 2008.

[13] H. Wang, R. J. Jaszczak, and R. E. Coleman. A new composite model of objects for monte carlo simulation of radiological imaging. *Phys Med Biol*, 38(9):1235–1262, Sep 1993.

[14] Roy Wollman and Nico Stuurman. High throughput microscopy: from raw images to discoveries. *J Cell Sci*, 120(Pt 21):3715–3722, Nov 2007.

Part IV. Screenshots for STSE image digitization

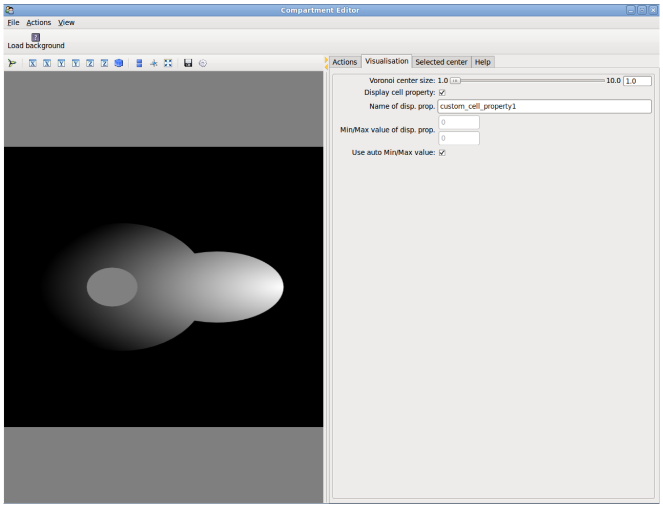

Fig. 11: Fus3^{PP} image (`shmoo_05.png`) loaded into STSE Compartment Editor.

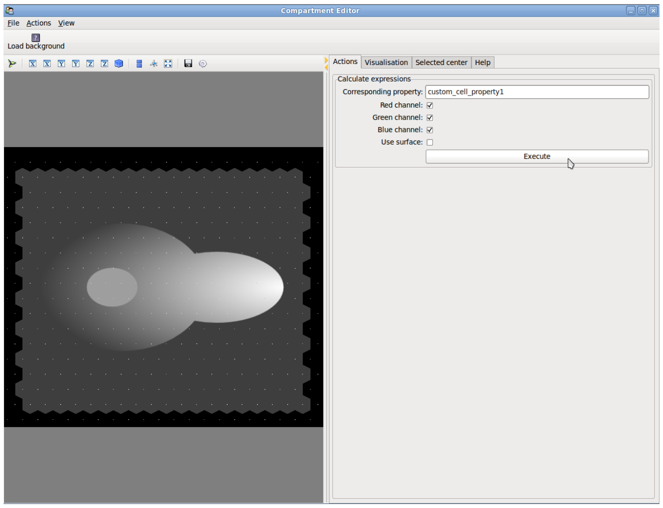

Fig. 12: Compartment geometry edition with STSE Compartment Editor.

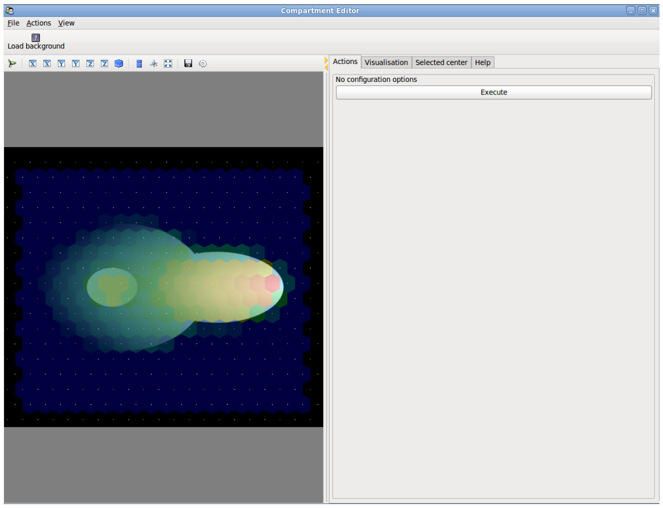

Fig. 13: Calculate expression for Fus3^{PP} inside STSE Compartment Editor.

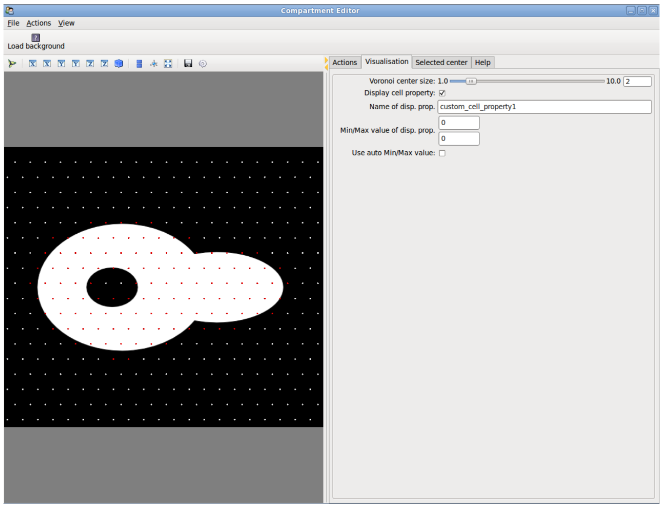

Fig. 14: Define cell type (here: cytoplasm) in STSE Compartment Editor.

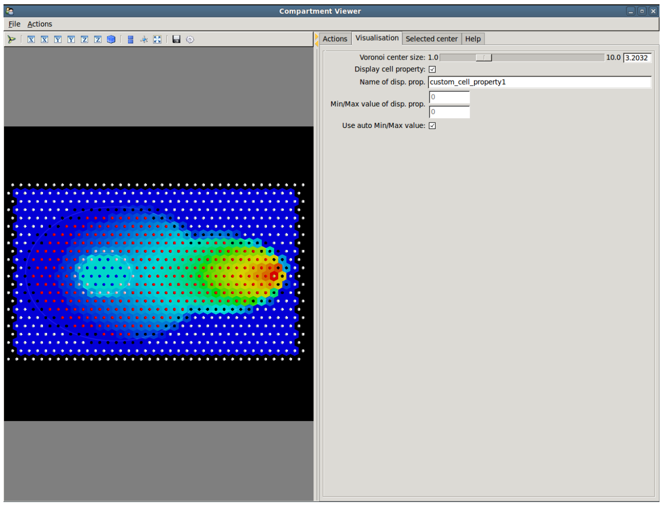

Fig. 15: Fus3^{PP} signal intensity acquired into STSE data structure with cell types set in previous steps. /
